# Supplementary material for: Atomic scale symmetry and polar nanoclusters in the paraelectric phase of ferroelectric materials
Source: Nat Commun. 2021 Jun 9;12:3509. doi: 10.1038/s41467-021-23600-3 (PMC8175364; doi:10.1038/s41467-021-23600-3)
Supplement: Supplementary file 1 — Supplementary Information [file 41467_2021_23600_MOESM1_ESM.pdf]

Supplementary Information

Atomic scale symmetry and polar nanoclusters in the paraelectric phase of ferroelectric materials

Andreja Bencan[1], Emad Oveisi[2], Sina Hashemizadeh[3,4], Vignaswaran K. Veerapandiyan[5], Takuya Hoshina[6], Tadej Rojac[1], Marco Deluca[5], Goran Drazic[7], Dragan Damjanovic*[,3]

1) Electronic Ceramics Department, Jozef Stefan Institute, 1000 Ljubljana, Slovenia,

2) Interdisciplinary Center for Electron Microscopy, Ecole Polytechnique Fédérale de Lausanne, 1015 Lausane, Switzerland,

3) Group for Ferroelectrics and Functional Oxides, Institute of Materials, Ecole Polytechnique Fédérale de Lausanne, 1015 Lausanne, Switzerland

4) Present address: Foundation for Research on Information Technologies in Society (IT'IS), Zeughausstr. 43, 8004 Zurich, Switzerland

5) Materials Center Leoben Forschung GmbH, Roseggerstrasse 12, 8700 Leoben, Austria

6) School of Materials and Chemical Technology, Tokyo Institute of Technology, Meguro, Tokyo, Japan

7) Department of Materials Chemistry, National Institute of Chemistry, 1000 Ljubljana, Slovenia

*email of the corresponding author: dragan.damjanovic@epfl.ch

Table of Contents

1. Materials preparation, average structure and defects
2. Experimental details of HAADF/ABF imaging, EDXS, $(Ba,Sr)TiO_3$ image simulations and displacement measurements
3. $BaTiO_3$ simulations and displacement measurements
4. Quantitative HAADF STEM analysis of atom column intensities and average chemical composition of $(Ba,Sr)TiO_3$
5. Influence of overlapping polar clusters along the viewing direction on displacement measurements
6. Evidence of strain associated with polar nanoclusters
7. Stability of the samples under the electron beam during HDAAF measurements and limits of vacancy concentration detection

1. Material preparation, average structure and defects

Preparation and structure. For more details on samples preparation see refs.[1,2], which present processing conditions and results of detailed microstructural characterizations of samples, including by scanning electron microscopy. BST6040 samples were prepared from commercial barium titanate (0.2 μm) and strontium titanate (0.1 μm) powders (see subnote Defects below for manufacturer and purity information). The starting powders were dispersed in isopropanol according to stoichiometric amount, the obtained slurry was mixed and milled by ZrO_2 balls (5 mm diameter) for 24 hours using a planetary milling machine. After milling, the mixture was dried in a glass beaker on a hot plate at 358 K. Calcination of dried powder was performed at 1423 K for two hours with a heating and colling rate of 5 K/min. In the final step, solid-state pressed green samples were sintered in air at 1723 K for 4 hours with 5 K/min heating and cooling rates. $BaTiO_3$ starting powder was mixed in a ball mill for 24 hours before pressing into pellet which were sintered at 1723 K for 4 hours.[2,3] $BaTiO_3$ powder was used for STEM studies, after grinding and annealing at 1173 K for 2 hours (see also Methods).

The X-ray diffraction spectra, Supplementary Figure 1a, show that the average macroscopic structure of $(Ba_{0.6}Sr_{0.4})TiO_3$ (BST6040) is cubic perovskite at room temperature. The lattice parameter determined from XRD for BST6040 is ≈3.964 Å (see Ref. [4]), which is in good agreement with the values reported by other authors.[5] The corresponding data for $BaTiO_3$, which possesses tetragonal perovskite structure at room temperature, can be found in Ref.[2]. The dielectric permittivity of BST6040 as a function of temperature, Supplementary Figure 1b, exhibits expected sequence of phase transitions[6] while the thermal hysteresis indicates a first order character of all phase transitions. We have prepared several more compositions in the $(Ba_{1-x},Sr_x)TiO_3$ solid solution and the trend of the Curie temperature versus composition follows Vegard's law, indicating good mixture of Ba and Sr.[1] All these data corroborate microscopic results that Ba- and Sr-titanate precursors are well mixed and that Ba and Sr are homogeneously distributed over A-sites of the perovskite structure.

The macroscopic polarity in the paraelectric phase of $(Ba_{1-x}Sr_x)TiO_3$ and $BaTiO_3$ is a manifestation of presence of nano-size polar clusters. We have observed and systematically studied macroscopic polarity in dozens of $(Ba_{1-x}Sr_x)TiO_3$ samples, including end members $SrTiO_3$ and $BaTiO_3$, prepared from different precursors (e.g., sol-gel, carbonates, oxides and titanates), by different sintering conditions and with different grain size.[1–4,7] The macroscopic polarity was also observed in undoped single crystals of $BaTiO_3$,[1,4] proving that the macroscopic polarity and thus polar nanoclusters are a property of these materials and not specific samples. Majority of the samples we investigated were prepared at EPFL (Alberto

Biancoli and Sina Hashemizadeh) and some at Tokyo Institute of Technology (Prof. Takuya Hoshina). All samples used in this study were prepared at EPFL and are representative samples of the general behaviour.

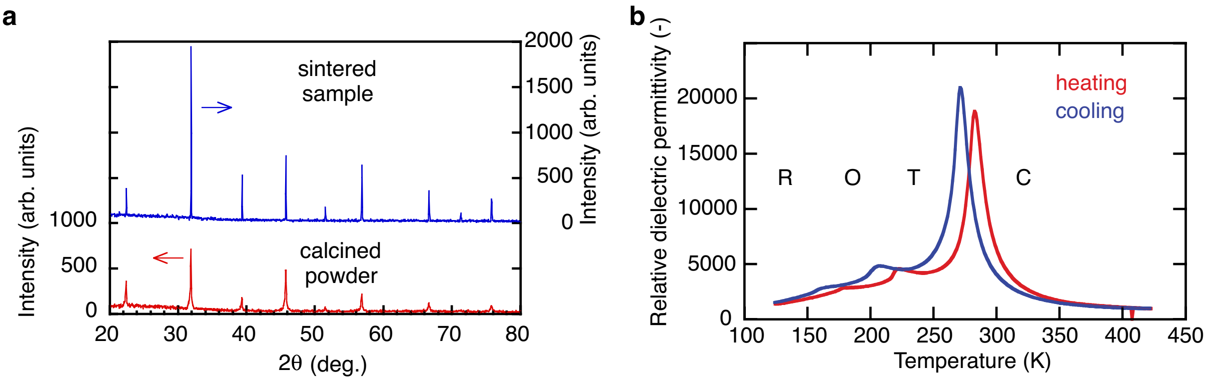

Supplementary Figure 1. Average macroscopic structure. **a** X-ray diffraction spectrum of $(Ba_{0.6}Sr_{0.4})TiO_3$ powder and sintered sample, measured at room temperature, showing macroscopic perovskite cubic structure. **b** Dielectric permittivity of BST6040 as a function of temperature. C, T, O and R indicate cubic, tetragonal, orthorhombic and rhombohedral phase, respectively.

Defects. In this study we used electronic grade barium titanate and strontium titanate powders from Inframat Advanced Materials, with 99.95% purity (metal basis) declared by the manufacturer. Manufacturer's data indicate that impurities in $BaTiO_3$ include: Ca<0.001%, Fe<0.001%, K<0.001%, Mg<0.001%, Na<0.001%, Sr<0.0015%. In $SrTiO_3$, which was used for preparation of BST, declared impurities include Ca<0.008%, Fe<0.006%, K<0.005%, Mg<0.002%, Na<0.006%. Concentration of other elements is not reported.

The maximum 0.05% concentration of impurities does not include concentration of vacancies of oxygen, barium and titanium, which are produced during high temperature processing or to compensate acceptor or donor dopants which are part of the quoted 0.05% impurity concentration. The actual concentration of defects is thus higher than concentration of declared impurities, but considering the low declared concentration of the most common impurity elements (see above) the defect concentration should still be on the order of 0.05%. This estimate of defects concentration agrees well with the value of the dielectric loss, which ranges from <0.01 at 100 kHz to 0.02 at 100 Hz[1,2] and Curie temperature, T_C, (≈400 K[1] vs 408 K in undoped single crystal[4]), both of which are dependent on defects concentration, see for example Rf.[8,9]

Let us assume that the concentration of defects is 0.05%. For $BaTiO_3$, a concentration of 0.05% (relative to either A- or B-site of the perovskite cell) translates to a concentration of defects of about $7.5x10^{18}$ cm^{-3} (density of $BaTiO_3$=6.02 g/cm^3; molecular weight=233.2 g/cm^3, one formula unit per unit cell). This is about 5 foreign in 10,000 host atoms (or 1 in 2,000). Actual defects are isovalent impurities which exert strain on the lattice through ionic size difference and aliovalent defects (impurities and vacancies needed for charge compensation) that exert influence both due to size and charge difference. Defects are distributed in three dimensions and not linearly, so in average one defect is separated from a neighbouring defect by about 12-13 unit cells or about 5 nanometers (in all three dimensions). The calculation is based on a cubic unit cell of 0.4 nm and one defect per 12.6x12.6x12.6 cells (≈2,000 cells). Note that even if the defect concentration is ten times lower (1 defect per 20,000 host atoms), this would still be about 1 defect per 27 unit cells, or 1 defect every 10 nm, close to the size of polar chains as estimated by Lambert and Comes.[10] In other words, since the polarization within nanoclusters is a correlated effect, freezing of ionic displacements in one unit cell may have an effect on the dipole direction in the neighbouring cells, freezing polarization orientation in the whole nanocluster.

2. Experimental details of HAADF/ABF imaging, EDXS, (Ba,Sr)TiO_3 image simulations and displacement measurements

The method for displacement calculation. STEM imaging along the $[110]_{pc}$ direction was carried out on a Jeol ARM 200 CF operated at 200 kV. HAADF and ABF detectors were used simultaneously at 68–180 and 10–16 mrad collection semi angles, respectively. Beam convergence semi-angle was 24 mrad. To minimise the influence of the specimen drift and scanning irregularities on the atomic column positions HAADF and ABF images were taken as a stack of 20 images; each frame was taken with pixel time of 1.6 μs (2 s per frame) using DigiScan Stack Acquisition Tool DigitalMicrograph script by Bernhard Schaffer.[11] After the acquisition all images in the stack were aligned using cross-correlation and averaged to obtain low-noise, good quality STEM images using Stack Alignment DigitalMicrograph script written by D.R.G. Mitchell.[12]

HAADF and ABF images used for Ti vs (Ba,Sr), O vs (Ba,Sr) and O vs Ti displacement measurements were taken in $[110]_{pc}$ zone axis, represented in terms of pseudo cubic parameters (see Supplementary Figure 2). Coordinates of atomic column positions were determined using 2D Gaussian fit.

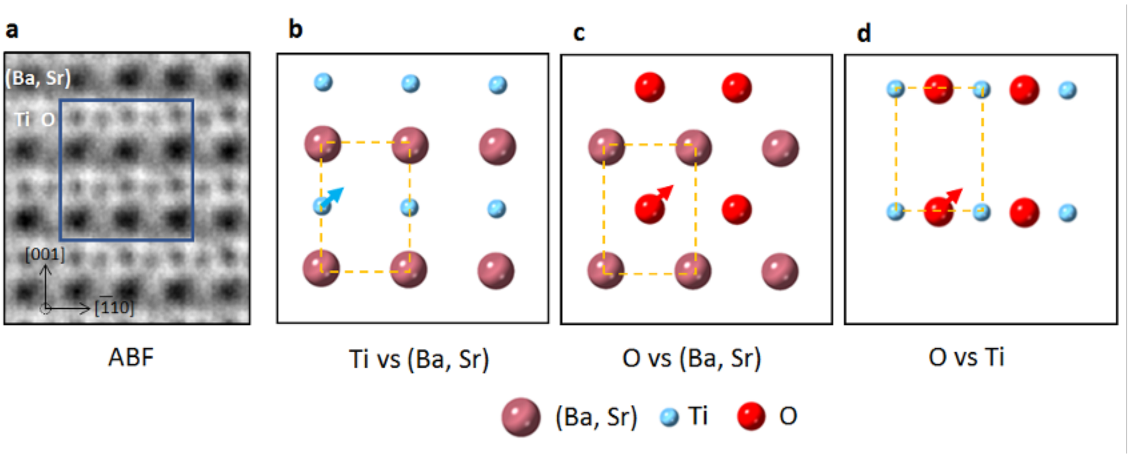

Supplementary Figure 2. Atomic displacement calculation. a ABF image of (Ba,Sr)TiO_3 in $[110]_{pc}$ zone axis where Ba/Sr (most intense large dark circles), Ti (directly below/above (Ba,Sr)) and O (diagonally from (Ba,Sr)) columns are seen. Blue rectangle indicates the area where the methodology of atom columns displacements is explained. **b**, **c** and **d** represent schematics of how individual atom columns displacements were measured. The reference frame was set as indicated by orange dashed rectangle (positions in (Ba,Sr) and Ti sub-lattices) and the displacements were measured as a deviation from the ideal cubic positions (in the case of Ti vs (Ba,Sr) this position is the middle point at the line between two (Ba,Sr) columns, in the case of O vs Ba this position is the middle point inside the rectangle forming four (Ba,Sr) columns and in the case of O vs Ti this positions is the middle point at the line between two Ti columns). The measured atom column displacements were defined with two parameters, the size of the displacements (in pm) and the angle of the displacements (0° –360°).

STEM imaging along the $[001]_{pc}$ direction was carried out on a Thermo Scientific Titan Themis 60-300 at 300 kV. HAADF and ABF images were simultaneously acquired at 90–170 and 9–18 mrad collection semi angles, respectively. In order to reduce statistical image noise and correct linear and non-linear scan distortions, each presented image is the average of a series of rapidly acquired images (approximately 100 frames with 512x512 pixels with 23.5 pm pixel size and a frame time of 500 msec) that underwent rigid and non-rigid alignment using the Smart Align software.[13]

The central position associated with each atomic column was identified on the aligned STEM images by using a two-dimensional Gaussian fitting in the StatSTEM software package.[14] Displacements for the B-site columns were determined by measuring their displacement relative to the mass centre of the four nearest neighboring A-site columns.

Atomic resolution energy-dispersive X-ray spectroscopy was performed on the same machine using a Super-X EDX system comprising four silicon drift detectors, and Velox acquisition software. Spectrum images with 256x256 pixels (pixel size 16.6 pm) and 100 ms frame time (100 frames) were acquired. Elemental maps were acquired using the Ba-Lα,

Sr-Lα, and Ti-Kα signals. A combination of Gaussian and Weiner filtering was applied on the elemental maps presented in Figure 3 b,c.

Effect of sample mistilt and thickness on displacement measurements, (Ba,Sr)TiO_3 simulations, and determination of the error in the relative atomic displacement calculations. The effect of sample mistilt and thickness on atom displacement measurements from ABF images has been reported in the literature.[15–17] Having made special effort to tilt the sample in the exact zone axis, EELS was used to determine the sample thickness. Nevertheless, there is a possibility that due to thermal effects the sample bends to some extent. Below we explain in details our methodology to test for potential mistilt of samples.

We simulated HAADF and ABF images of BST6040 using quantitative image simulation code (QSTEM)[18] with a multi-slice method and frozen phonon approximation. For simulations we used the same instrumental parameters as for experimental imaging (0 defocus, 24 mrad beam convergence semi - angle, 200 kV acceleration voltage). Thermal diffuse scattering (TDS) was included in simulations, so 7-30 repetitions of calculations per one image were used where the atom positions were varied in the interval set by estimated Debye-Waller factors for each calculation.

In order to examine the influence of the sample mistilt and thickness on displacement measurements, we performed simulations of HAADF and ABF images of *Pm*-3*m* cubic structure (using parameters from Inorganic Crystal Structure Database file ICSD #90006) in $[110]_{pc}$ zone axis for different thicknesses and at 0 ° and 0.5° specimen mistilt angles. Using 2D Gaussian fit we extracted the exact (Ba,Sr), Ti and O atom column positions and calculated Ti vs (Ba,Sr), O vs (Ba,Sr) and O vs Ti displacements (See Supplementary Figures 3 - 5 and Supplementary Table 1).

As shown in Supplementary Figure 3 the influence of the sample thickness up to 25 nm on the measured displacements of Ti vs (Ba,Sr) in *Pm*-3*m* BST6040 at zero specimen mistilt is negligible regardless of whether HAADF or ABF images were used. The maximum displacement up to 2 pm was obtained (see Supplementary Figure 3 and Supplementary Table 1). The expected relative atomic displacements for the ideal cubic structure at zero tilt should be zero. Thus, the maximum displacement measured for the simulated cubic structure (~2 pm) defines the method's error.

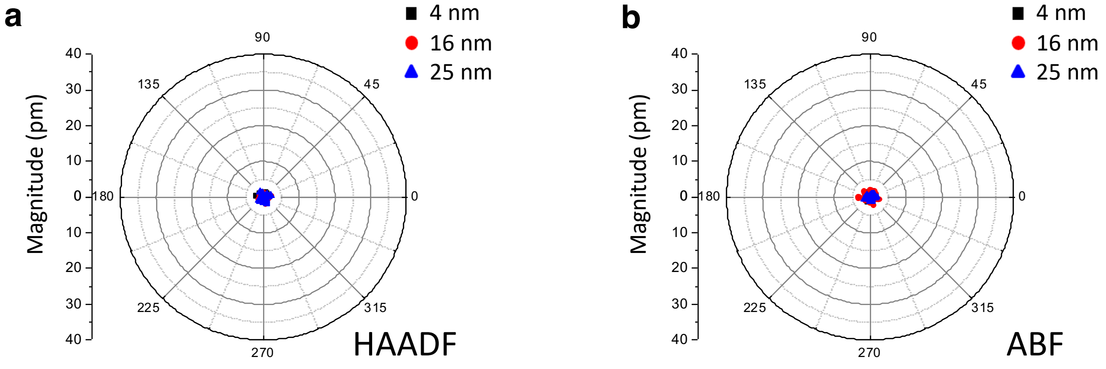

Supplementary Figure 3. Polar figures of Ti vs (Ba,Sr) displacements in *Pm*-3*m* BST6040 measured from simulated **a** HAADF and **b** ABF images of cubic symmetry at different sample thicknesses (4, 16 and 25 nm), at 0° sample mistilt angle.

The sample mistilt (0.5°) does not influence Ti vs (Ba,Sr) displacements measured from HAADF images at different thicknesses as shown in Supplementary Figure 4a. However, the sample mistilt strongly influences both magnitude and direction of displacements measured from ABF images across the examined thickness range as shown in Supplementary Figure 4b.

In Supplementary Table 1 average Ti vs (Ba,Sr) displacements at 0 ° and 0.5 ° sample mistilt measured from simulated HAADF and ABF images of cubic symmetry at different thicknesses are summarized.

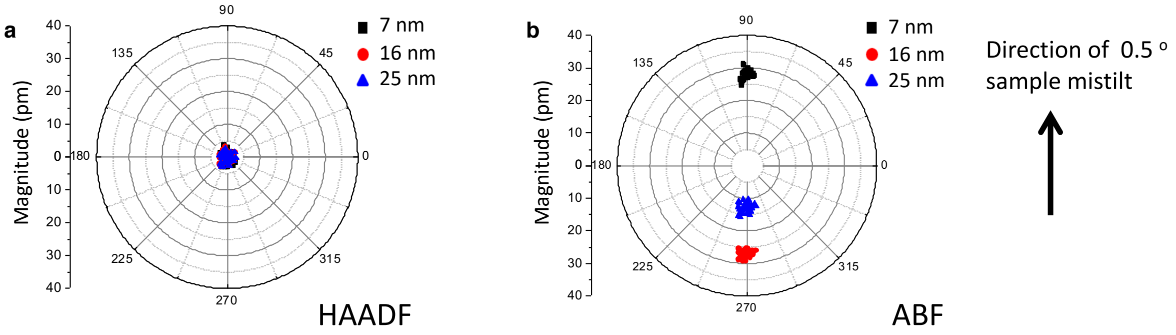

Supplementary Figure 4. Polar figures of Ti vs (Ba,Sr) displacements in *Pm*-3*m* BST6040 measured from simulated **a** HAADF and **b** ABF images at different sample thicknesses (7,16, 25 nm), at 0.5° sample mistilt angle.

Supplementary Table 1. Average Ti vs (Ba,Sr) displacements with one standard deviation, at 0° and 0.5° sample mistilt measured from simulated HAADF and ABF images of cubic symmetry, at different thicknesses.

Sample thickness / mistilt (nm / °)	Displacements Ti vs (Ba,Sr) from HAADF image (pm)	Displacements Ti vs (Ba,Sr) from ABF image (pm)
4 / 0	0.9 ± 0.4	0.9 ± 0.4
16 / 0	1.0 ± 0.5	1.3 ± 0.6
25 / 0	1.0 ± 0.5	0.9 ± 0.4
7 / 0.5	2.0 ± 0.9	28.0 ± 1.3
16 / 0.5	1.6 ± 0.9	27.2 ± 1.0
25 / 0.5	1.6 ± 0.8	13.1 ± 1.3

Importantly, in the case of 0.5° sample mistilt the amplitude and direction of all interatomic displacements (Ti vs (Ba,Sr), O vs (Ba,Sr) and O vs Ti) in *Pm*-3*m* BST6040 determined from simulated ABF images strongly depend on sample thickness, as can be seen from Supplementary Figure 5.

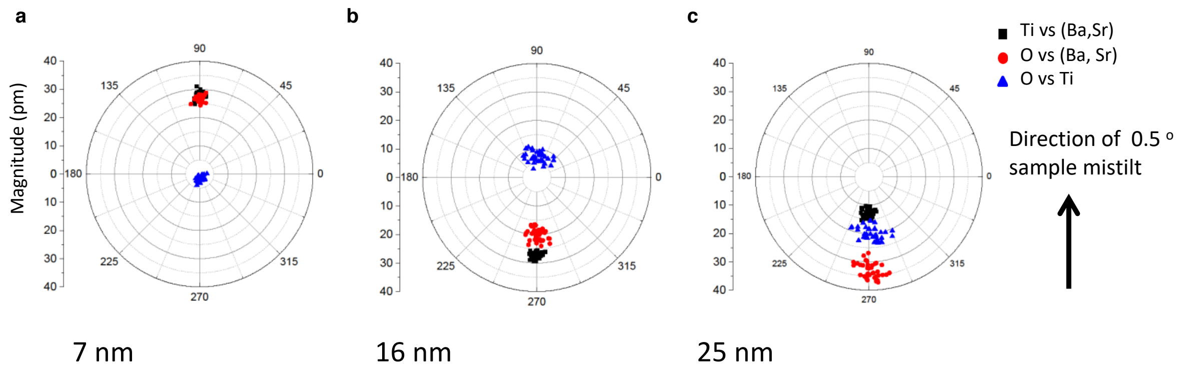

Supplementary Figure 5. Polar figures of Ti vs (Ba,Sr), O vs (Ba,Sr) and O vs Ti displacement measured from ABF images at **a** 7 nm, **b** 16 nm and **c** 25 nm sample thicknesses, at 0.5° sample mistilt angle.

To illustrate that our experimental ABF images are not affected by the specimen mistilt we analysed two regions with different thicknesses and performed displacement measurements as shown in Supplementary Figure 6. We can see that we obtain practically identical displacements in magnitude and direction, indicating negligibly sample mistilt. As explained in the main text (see Fig. 2) the analysed area can be ascribed to non-cubic symmetry.

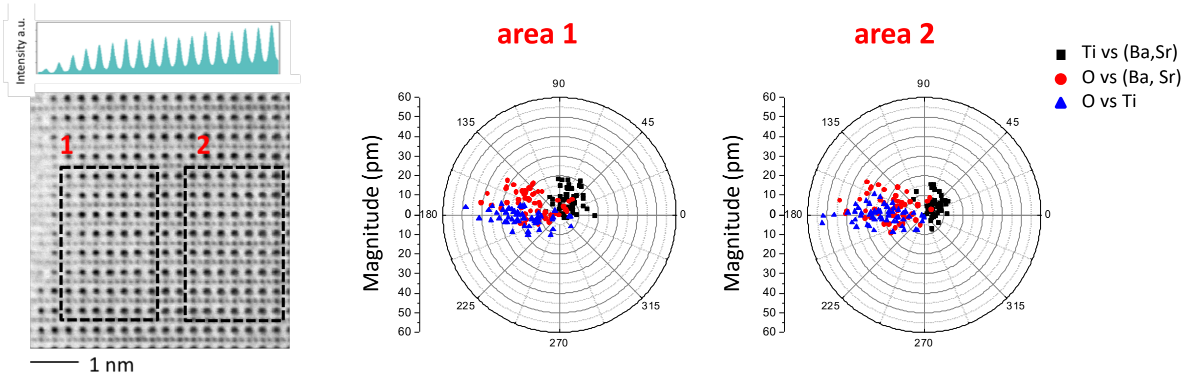

Supplementary Figure 6. ABF image along $[110]_{pc}$ zone axis with displacements of Ti vs (Ba,Sr), O vs (Ba,Sr) and O vs Ti taken from two marked areas with different thickness as indicated by intensity profile taken from corresponding HAADF image shown in Figure 2.

We note that determined displacements in BST6040 (considering difference in the reference point with respect to which displacement were measured) are comparable to those calculated in reverse Monte Carlo refinements from multiple measurement techniques. For example, that study shows that addition of Sr causes off-centering of Ti in the range of 12 to 16 pm.[19]

3. $BaTiO_3$ simulations and displacement measurements

To correlate the experimental atom column displacements with theoretical displacements we simulated ABF images of $BaTiO_3$ as explained for simulations of BST6040 images in Supplementary Note 2. We created cubic *Pm*-3*m* (ICSD #27970), orthorhombic *Amm*2 (ICSD #161341), tetragonal *P*4*mm* (ICSD #154343), and rhombohedral *R*3*m* (ICSD #73635) structural models of 5 nm thickness. Using these models we then simulated the ABF images in $[110]_{pc}$ zone axis (in pseudo-cubic notation) and using 2D Gaussian fit we extracted the exact Ba, Ti and O atom column positions and calculated Ti vs Ba, O vs Ba and O vs Ti displacements. In Supplementary Figure 7 the displacements are compiled in the composite polar plots, where the directions of displacements are more clearly delineated.

The error in the displacement determination is estimated to about 2 pm from calculated displacements in the simulated cubic structure. While in the cubic structure all displacements are by definition zero, the method accurately predicts split of atomic displacements within tetragonal, orthorhombic and rhombohedral phases.

Experimental atomic displacements were first measured on $BaTiO_3$ sample at room temperature (RT) (i.e., in the tetragonal phase) along $[110]_{pc}$ zone axis, Supplementary Figure 8. The spread of atomic shits is more than twice larger in the experimental than in modelled

images; the maximal standard deviation in the experimental case is 7 pm versus 3 pm in the modelled structure indicating disorder in the sample (compare Supplementary Figure 7 and 8). This disorder could be indicative of a non-negligible concentration of cationic and O vacancies, whose presence would distort the structure. Alternatively, it could be a consequence of a ferroelectric domain wall within the examined area. Supplementary Figure 9 shows data taken at 573 K (see the main text).

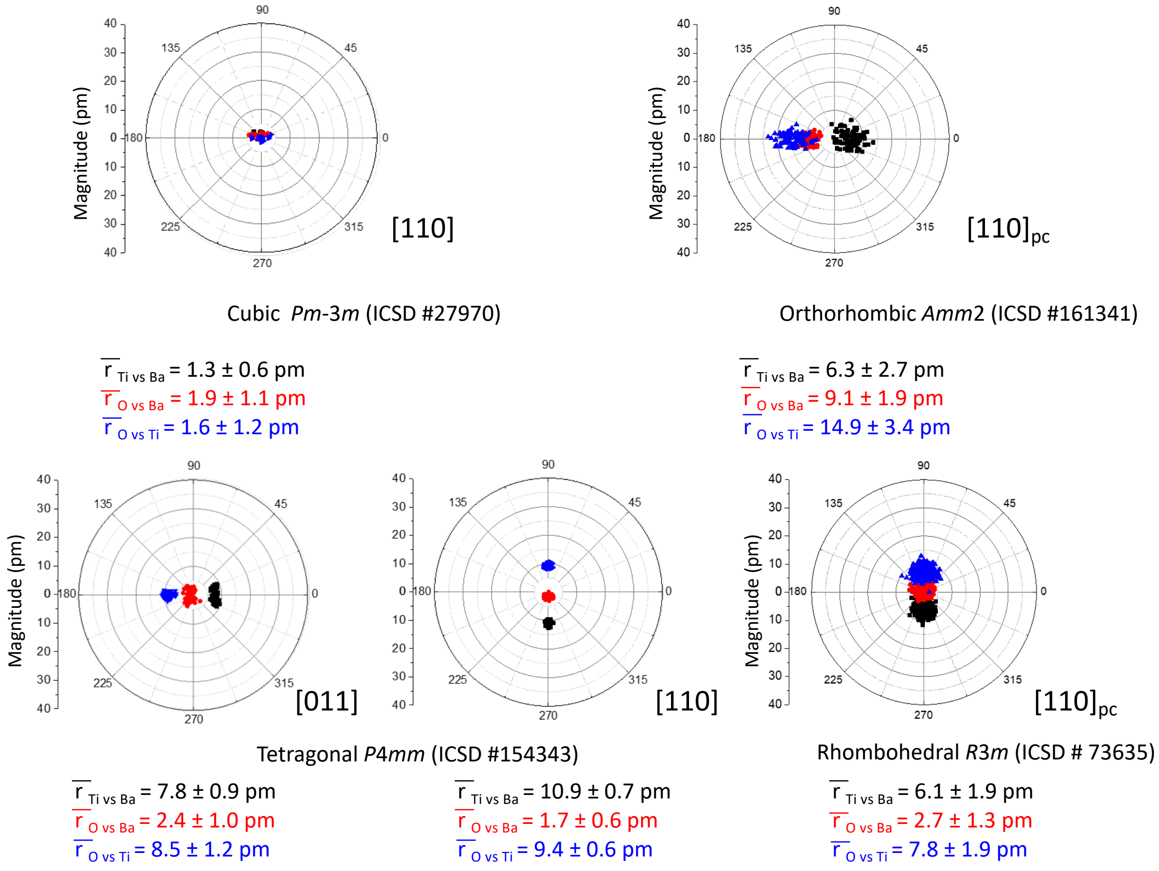

Supplementary Figure 7. $BaTiO_3$ simulations. Composite pole figures of different displacements for cubic, tetragonal, orthorhombic and rhombohedral symmetries with corresponding average absolute displacement values. As expected, in cubic symmetry displacements are around 0. In all other symmetries, displacements are split (Ti vs Ba and O vs Ti are in the opposite directions). In tetragonal and rhombohedral symmetries O vs Ba displacements are distributed around origin of the coordinate system, while in the orthorhombic symmetry O vs Ba displacements are centred away from origin of the coordinate system.

We note that the determined displacements are comparable to those predicted by Density Functional Theory (DFT) computations.[20–22] Results of some of available calculations show that, for example, addition of one oxygen vacancy in the unit cell of $BaTiO_3$ causes a displacement of Ba by 14 pm and displacement of O ions by 6 pm and 23 pm.[20] In another

study, addition of two Fe ions to cubic $BaTiO_3$ in 2x2x2 super cell, caused a tetragonal distortion of the unit cell by 21 and 30 pm.[21] In yet another study with co-doping of $BaTiO_3$ with Ni and Fe, Ni causes displacement of O atoms by 12 pm, while an oxygen vacancy displaces neighbouring Ti atoms by 28 and 22 pm.[22] It should be mentioned that results of those DFT computations are based on many approximations: for example, cubic phase of $BaTiO_3$ is not the ground phase, a fully relaxed phase of $BaTiO_3$. External constraints must be introduced to keep the structure cubic and results depend on the choice of the supercell and other parameters[22,23]

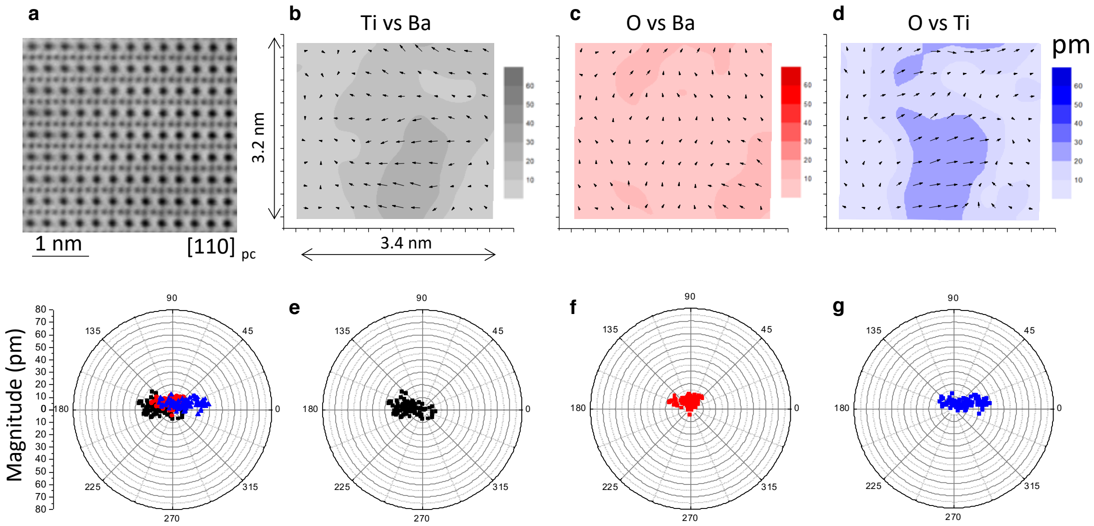

Supplementary Figure 8. $BaTiO_3$ experimental results at RT before in situ heating. **a** ABF image along $[110]_{pc}$ zone axis taken at RT (before in situ experiment) with a composite pole plot; **b**-**d** maps of displacements (arrows represent the direction of the displacements and their magnitude in pm - see colour scale on the right) with corresponding **e**-**f** pole figures. The average values of the relative atomic displacements measured over the whole image are 12±6 pm for Ti vs Ba, 8±4 pm for O vs Ba, and 13±7 pm for O vs Ti. Due to antiparallel displacement of Ti vs Ba compared to O vs Ti and displacements of O vs Ba around centre, the overall structure seems, as expected, to be tetragonal.

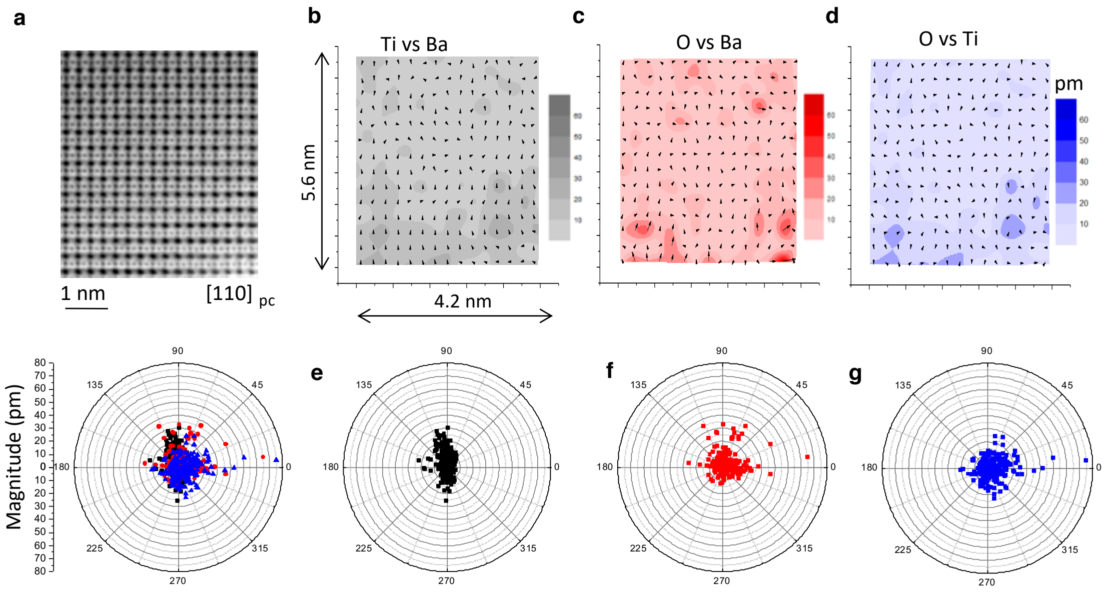

Supplementary Figure 9. $BaTiO_3$ relative atomic displacements at 573 K. a ABF image along $[110]_{pc}$ zone axis taken at 573 K with a composite pole plot. **b**-**d** Maps of displacements (arrows represent the direction of the displacements and their magnitude in pm - see colour scale on the right) with corresponding **e**-**f** pole figures. The average values of the relative atomic displacements measured over the whole image are 10±6 pm for Ti vs Ba, 10±8 pm for O vs Ba, and 11±9 pm for O vs Ti. Splitting of displacements is much smaller than at 200°C indicating that symmetry is approaching cubic.

4. Quantitative HAADF STEM analysis of atom column intensities and average chemical composition of $(Ba,Sr)TiO_3$

To determine local stoichiometry of BST6040, normalized (Ba,Sr)-column intensities were measured using the approach by LeBeau & Stemmer[24] where the intensity of the background and individual atom columns were measured from the HAADF images at non-saturating settings of brightness and contrast. The detector background intensity was subtracted from the intensity of each pixel in experimental images. (Ba,Sr) and Ti column intensities were extracted form HAADF images as intensity peak integral, integrated within roughly two sigma by approximating a 2D Gaussian-type peak. The final values of individual atom-column intensities were normalised to the highest column intensity in the analysed area to cancel the influence of HAADF detector amplification (for details see also ref. [25]). A quantitative estimate of the intensity distribution of A sites due to chemical fluctuations in BST6040 was then obtained by simulating HAADF image of a cubic structural model of 10 nm thickness in $[110]_{pc}$ zone axis image (for simulation details see Supplementary Note 2) with randomly distributed

Ba and Sr atoms in 60/40 ratio. For each A site, using random generator, Ba or Sr atom were assigned in 0.6/0.4 probability ratio.

We get from the model in $[110]_{pc}$ orientation that the expected average intensity for a random distribution of $Ba_{0.60}$ and $Sr_{0.40}$ on A-site is 0.88±0.05. The intensity is expressed in relative terms, with intensity 1 being assigned to a column with the highest intensity. The average experimental intensity value obtained is 0.94±0.02 for a 20 nm thick BST6040 sample (Supplementary Figure 10), and 0.90±0.02 for a 5 nm thick sample (not shown). These values are within the experimental error of a perfectly random distribution of Br and Sr with 60/40 stoichiometry.

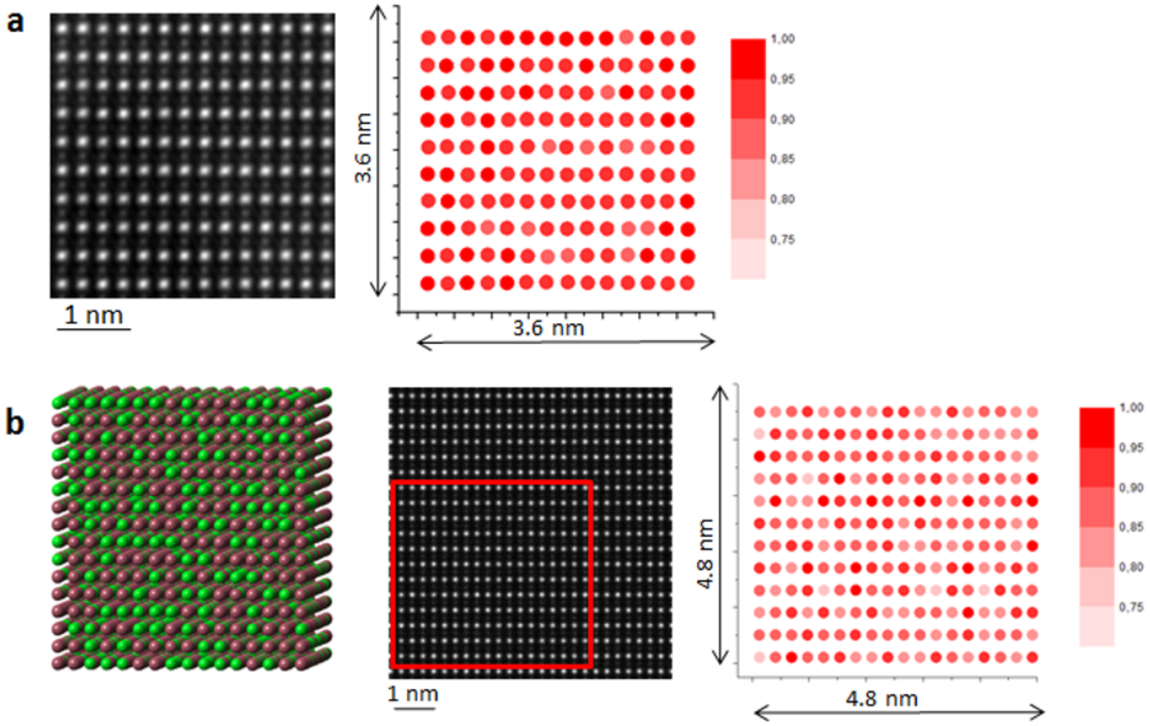

Supplementary Figure 10. Average chemical composition. a Experimental HAADF image of BST (simultaneously taken with ABF image shown in Figure 2) in $[110]_{pc}$ zone axis with the corresponding normalized intensity distribution map of (Ba,Sr)-columns. Average relative intensity of (Ba,Sr) column intensity is 0.93±0.02. **b** A part of the model showing only Ba (brown) and Sr (green) atoms, with the simulated HAADF image and corresponding normalized intensity distribution map of (Ba,Sr) columns. Chemical homogeneity (average relative intensity of Ba, Sr-site atom columns) estimated for a 15 nm thick BST6040 model where Ba and Sr randomly occupy A sites is 0.88±0.05. The results indicate that there is no significant departure in the experimental data from the random distribution of Ba and Sr atoms with 60/40 stoichiometry.

We finally consider the possibility of concentrated distribution of Ba atoms in one part of a column (still keeping the stoichiometric Ba/Sr ratio within that column). Even if this does happen in one column it is unlikely to occur in the majority of the columns within a given polar nanocluster. If the polarity is to be explained by such concentration of Ba within one part of the column, that trend would have to repeat for majority of columns in all nanoclusters. Since the intensity signal is dominated by the first ~5 nm thick layer, this would mean that there is a layer within the material in which Ba is concentrated over the whole examined region. But then, it would be equally likely that there should be layers where Sr is concentrated and we do not see either of these cases.

5. Influence of overlapping polar clusters along the viewing direction on displacement measurements

To estimate the effects of embedded clusters on displacement measurements, two situations were considered: i) a polar tetragonal volume embedded into a cubic matrix (Supplementary Figure 11), and (ii) four alternating polar and cubic (non-polar) regions (Supplementary Figure 12).

In the first model, a 4 nm-thick stack of BST6040 with tetragonal structure is embedded into a cubic volume, of the same composition, Supplementary Figure 11. Simulated ABF image of the model in [110] zone axis gives O vs (Ba,Sr) and O vs Ti displacements of about 12.5±1.1 pm, which is close (within experimental accuracy) to the assigned displacements of 14.7 pm in the modelled tetragonal BST6040.

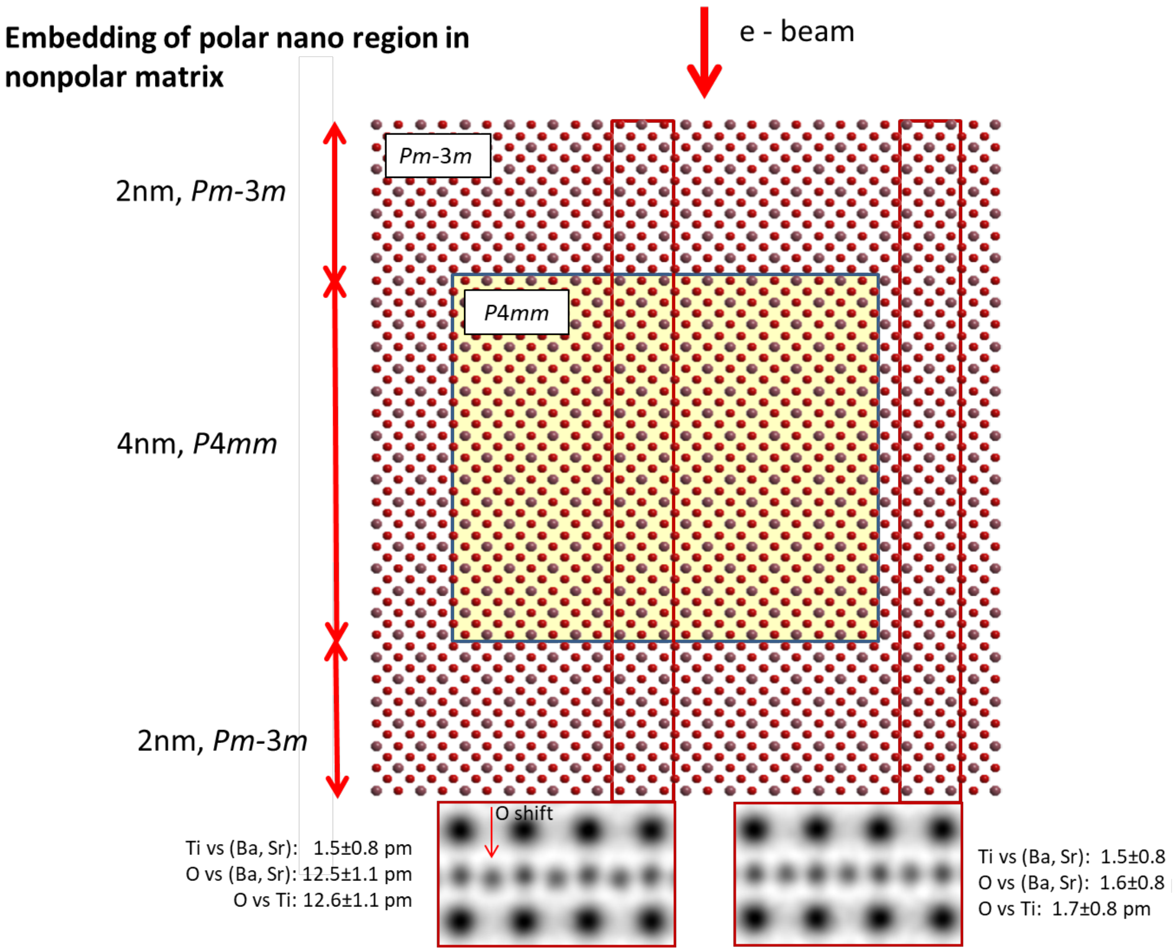

Supplementary Figure 11. Simulated displacements in BST6040. Schematic of simulated tetragonal *P*4*mm* (ICSD # 188786) embedded volume in cubic *Pm*-3*m* (ICSD # 90006) matrix (with indicated direction of the electron beam (e-beam) and dimensions) and ABF images calculated from the model in overlapped (left) and pure cubic volumes (right). The displacements set for the tetragonal volume, calculated from the *P*4*mm* model are Ti vs (Ba,Sr) 0 pm, O vs (Ba,Sr) 14.7 pm, and O vs Ti 14.7 pm. Measured displacements in overlapped volume are Ti vs (Ba,Sr) 1.5±0.8 pm, O vs (Ba,Sr) 12.5±1.1 pm, and O vs Ti 12.6±1.1 pm, that is all within experimental error. In pure cubic phase displacements are, as expected, close to 0, i.e. Ti vs (Ba,Sr) 1.5±0.8 pm, O vs (Ba,Sr) 1.6±0.8, and O vs Ti 1.7±0.8 pm. Based on these results we estimate that the first up to ~6 nm-thick surface region (where the beam is entering) crucially influences the image formation and consequently the displacement measurements.

In the second situation, with alternating polar/nonpolar layers, we considered three cases, Supplementary Figure 12. In Cases 1 and 2, ABF images were simulated from the entire, 24 nm-thick volume, but with opposite electron beam direction, as indicated in the figure. In Case 3, ABF image was simulated only from the top 3 nm layer (see Supplementary Figure 12). For the three cases the average displacements are collected in the corresponding Table. If all parts of the sample in Cases 1 and 2 would contribute equally to the image we should obtain displacement values around 0, but this is not the case. For Ti vs (Ba,Sr), in Case 1, we can state

that there are no displacements, if we ascribe the values of ~6 pm to the experimental error. O vs (Ba,Sr) and O vs Ti are almost doubled compared with displacement in individual tetragonal region (~12 pm, see Case 3). Obviously, opposite polarization does not cancel out; the main information is coming from the top region of the sample. The same conclusion can be made from Case 2, where all displacements are close to zero, because the cubic phase, where electrons enter the sample, prevails. In Case 3, where the sample is thin and has tetragonal symmetry, we measured the expected values (~0 for Ti vs (Ba,Sr) and ~12 pm for O vs (Ba,Sr) and O vs Ti).

Top few nm of the sample (where the electron beam is entering) has the largest effect on the image and consequently on the measured displacements. So even in thicker samples (20 nm) we may determine the presence of polar nano-regions if they are at the top part of the sample. In thicker samples the measured displacements are overestimated but their direction is correctly determined.

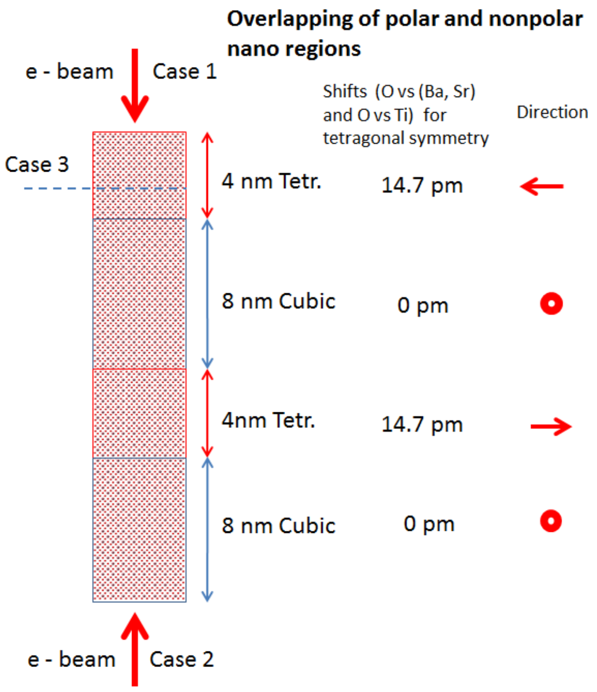

	Ti vs (Ba, Sr) (pm)	O vs (Ba, Sr) (pm)	O vs Ti (pm)
Case 1	5.7 ± 0.8	21.6 ± 1.8	27.0 ± 1.8
Case 2	3.6 ± 2.8	3.6 ± 1.9	4.6 ± 2.4
Case 3	0.8 ± 0.6	11.9 ± 1.3	12.0 ± 1.1

Supplementary Figure 12. Influence of thickness and overlapping layers on displacement determination. A model was constructed where 4 layers of alternating tetragonal and cubic phases were placed across a 24 nm-thick sample. The polarizations of two tetragonal areas were in opposite directions, as marked by horizontal red arrows. Zero polarization in the cubic phase is indicated by the red circle. Based on the model, three different ABF images were simulated. In Case 1 the electron beam was entering the sample from top to bottom through the whole thickness (24 nm). In Case 2 the electron beam was entering from bottom to top through the whole thickness, and in the Case 3 the electron beam was entering as in Case 1 but the image was simulated for the first 3 nm-thick volume.

To summarize, based on these results (Supplementary Figure 11 and 12) we can estimate that the first ~5 nm thick surface region (where the beam is entering) crucially influences the image formation and consequently the displacement measurements.

6. Evidence of strain associated with polar nanoclusters

We present two independent results, Supplementary Figure 13 and 14, as evidence for strain associated with polar nanoclusters.

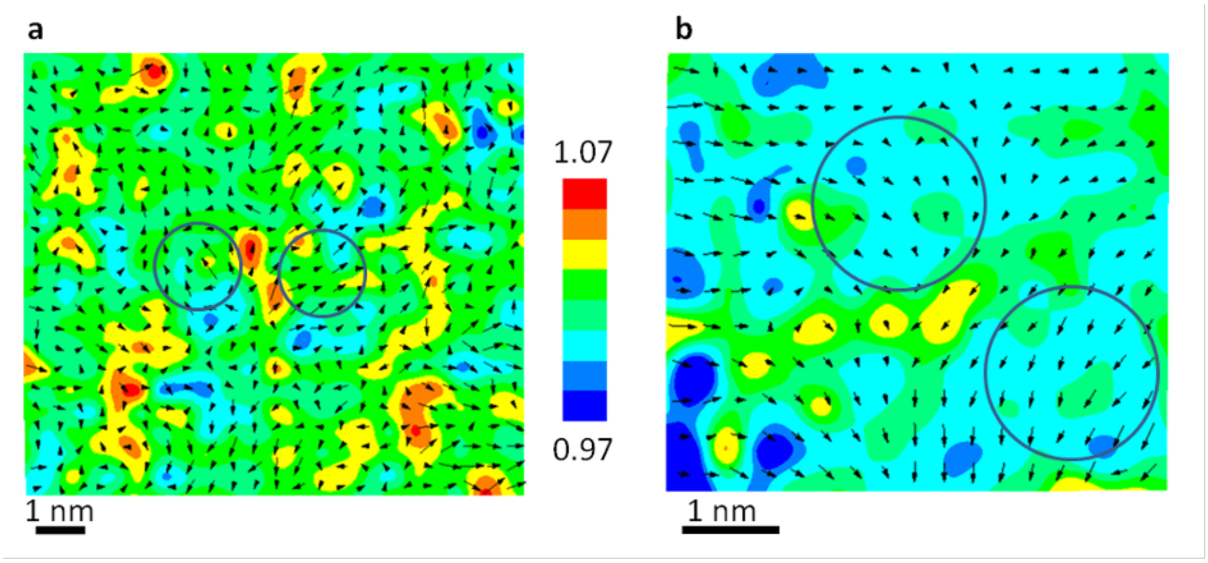

Supplementary Figure 13. Lattice strain distribution. a Lattice-strain map of BST6040 along $[001]_{pc}$ zone axis (from HAADF image in Figure 1a)**.** The lattice strain distribution was determined by extracting the horizontal (x) and vertical (y) (Ba,Sr) lattice parameters and is represented by the x/y ratio colour map. The map of the x/y ratio is superimposed on corresponding Ti vs (Ba,Sr) displacements, represented by orientation and length of the associated arrows. A correlation between the change of displacement direction and strain magnitude is visible. Maximal strains are observed at the interfaces between polar regions (as indicating by circles), suggesting that each region with coherent displacement of atoms is associated with specific strain distribution; **b** Lattice-strain map of $BaTiO_3$ along $[110]_{pc}$ zone axis (from ABF image in Figure 4a). The map of x/y ratio is superimposed on corresponding Ti vs Ba displacements at 200°C. A correlation between change of displacement direction and strain magnitude can be noticed. Polar regions are indicated by circles.

The complex elastic modulus *E* (mechanical stiffness or storage modulus and loss) was measured in the single cantilever mode with a Perkin-Elmer PYRIS Diamond Dynamic Mechanical Analyzer, with the temperature rate of 1–2 K/min, Supplementary Figure 14.

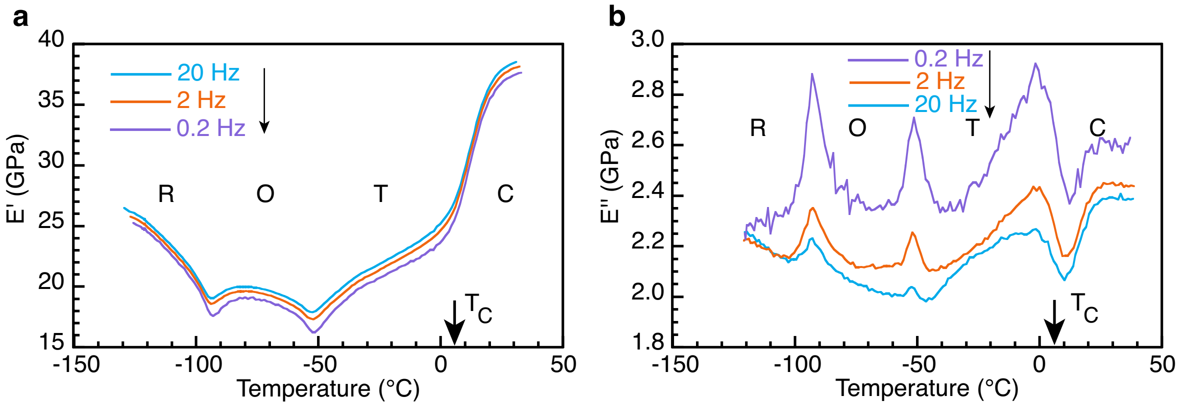

Supplementary Figure 14. Elastic modulus *E* as a function of temperature for BST6040. a Real, *E'*, and **b** imaginary component, *E''*, of *E*. The C, T, O and R mark cubic, tetragonal, orthorhombic and rhombohedral phase regions, respectively. T_C designates the Curie temperature. Compare with Supplementary Figure 1b. Two features are indicative of presence of elastically active nano-size objects within the material: (i) the large decrease of the elastic modulus *E'* in the cubic phase as the temperature is decreased toward Curie temperature T_C, and (ii) the frequency dependence of *E'* and *E''* in the cubic phase.[26,27] In the ferroelectric phases the temperature dependence and relaxation of *E'* and *E''* are dominated by ferroelastic domains. These results corroborate interpretation of microscopic strain data shown in Supplementary Figure 13.

7. Stability of the samples under the electron beam during HAADF measurements and limits of vacancy concentration detection

To test whether a significant, noticeable number of vacancies in Ba columns could be produced by prolonged irradiation of the sample with electrons, we evaluated intensities of 25 Ba columns in HAADF images acquired at different beam irradiation times (2 s, 10 s , 20 s and 600 s). HAADF images were acquired at 68-180 mrad collection semi-angles, with a camera length of 8 cm, spot size 6C (97 pA beam current), i.e. the experimental conditions used in our study. The images presented in the manuscript were typically collected as a stack of 10 individual 512x512 pixel images with 2 s frame time (8 μs/pixel). In Supplementary Figure 15 intensities were measured on 1st, 5th and 10th frame of the stack. In the case of 600 s the whole area was irradiated under similar conditions (8 μs/pixel) for 10 minutes and then the image was taken. We quantified the Ba column intensities in the HAADF images; the intensities of the individual atomic columns in each image were normalized to the highest column intensity in image after 2 s of irradiation (assumingly representing column without defects) and the average experimental intensity ratio was calculated as shown in Supplementary Figure 15. Details of the quantitative HAADF analysis of the atomic column intensities are given in Supplementary Note 4.

From Supplementary Figure 15, it can be seen that there is no major variation in the average Ba column intensity with time; the variations in the results are within the measurement error. Therefore, we show that $BaTiO_3$ is stable under electron beam at experimental conditions employed in this study.

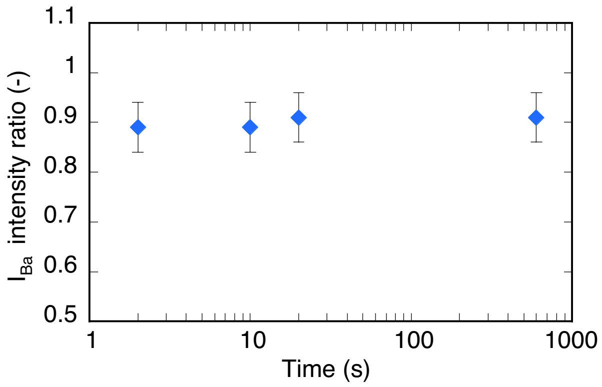

Supplementary Figure 15. Experimental average Ba-column intensity ratios (I_{Ba}) obtained after 2 s, 10 s, 20 s and 600 s of electron beam irradiation on $BaTiO_3$ heated in situ at 473 K. See the text for details. Bars represent 5% measurement error.

In addition, we irradiated the area with electrons for 10 min at 3.5 times higher beam current than used in our study, i.e., at spot size 3C (357 pA beam current) to demonstrate that the experimental conditions we used are far below those that would potentially damage the sample. Because the column intensity depends strongly on current used, we cannot directly compare the intensities. Therefore, we measured the intensity ratio between Ba and Ti columns for each current used. The average Ba/Ti intensity ratio before and after the experiment was 1.37±0.07 and 1.40±0.07, respectively. In the case that electron beam would induce generation of vacancies we expect that the number of generated vacancies would not be the same for Ba and Ti, i.e. the ratio Ba/Ti should change. Our results indicate that the number of vacancies formed in both Ba and Ti atomic columns even after using harsh TEM conditions is negligible.

We can therefore conclude that defects are not produced during STEM measurements under the experimental conditions used in this investigation.

To investigate the influence of the electron beam on the displacement measurements, we determine the Ti vs Ba displacements from the HAADF images obtained after 2 s, 10 s and 20 s of electron beam irradiation on ~4x4 nm polar cluster in $BaTiO_3$ sample that was in situ heated to 473 K. A total acquisition time of 20 s is the time required to obtain a high-quality STEM image, typically used for displacement analysis. As seen from Supplementary Figure

16 in all cases, the displacements Ti vs Ba indicate non-cubic symmetry and show similar directions and magnitudes. We therefore conclude that the nanoclusters are not formed or modified during observation of the sample in the TEM microscope, but are a property of the material.

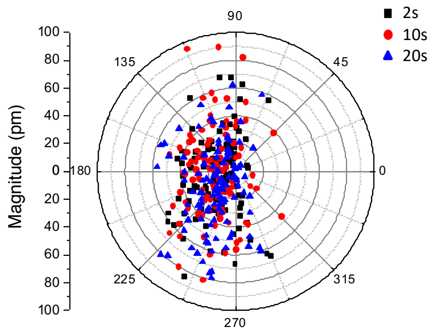

Supplementary Figure 16. Polar figure of Ti vs Ba displacements measured at different electron beam irradiation of a ~4x4 nm sized polar cluster in $BaTiO_3$ in situ heated to 473K. The average measured displacements after 2, 10 and 20 s are 30±18pm, 35±18 pm, 33±19 pm, respectively.

Finally, we simulated column intensities for different concentrations of vacancies, to see which concentration of vacancies may be detected under used experimental conditions. To correlate the normalized intensities of individual Ba-atom columns with the concentration of Ba vacancies inside each column, we compared the experimental intensities with calculated intensities. Calculations were performed using the QSTEM code[18] with multislice method and frozen phonon approximation. To include the influence of thermal diffuse scattering (TDS), 30 calculations per image were used. We created an *Pm*-3*m* $BaTiO_3$ structural model consisting of 8 x 8 x 40 unit cells in [001] zone axis using parameters close to experimental conditions. In Ba columns we introduced 2.5, 5, 10 and 20 at% Ba vacancies. Using these models we then simulated the HAADF images and extracted the corresponding Ba-column intensity ratios between columns with Ba vacancies and those with fully filled Ba positions. From Supplementary Figure 17 we see at least 20% of Ba vacancies are needed in order to get a statistically relevant result. The method is therefore unsuitable for detection of concentration of Ba-vacancies below about 10%. The experimental average intensity ratio is 0.91±0.05 which is in the range where no relevant conclusions can be made.

We thus conclude that, as expected, concentration of A-site vacancies in $BaTiO_3$ columns cannot be determined based on data taken in HAADF experiments.

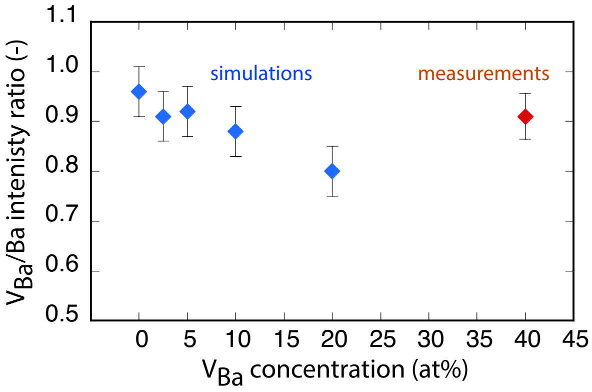

Supplementary Figure 17. Calculated Ba-column intensity ratio between columns containing Ba vacancies and those with fully filled Ba positions (V_{Ba}/Ba) as a function of Ba-vacancy concentration V_{Ba} (0, 2.5, 5, 10 and 20 at%), and experimental average intensity ratio obtained from five different polar nanoclusters in the $BaTiO_3$, heated in situ at 473K. Bars represent 5% measurement error defined as relative standard deviation of Ba column intensities without vacancies.

References

1. Biancoli, A. Breaking of the macroscopic centric symmetry in $Ba_{1-x}Sr_xTiO_3$ ceramics and single crystals. (PhD thesis; École Polytechnique Fédérale de Lausanne, Switzerland, 2014). doi:10.5075/epfl-thesis-6366.
2. Hashemizadeh, S. Origins of the macroscopic symmetry breaking in centrosymmetric phases of perovskite oxides. (PhD thesis; Ecole polytechnique fédérale de Lausanne, 2017). doi:10.5075/epfl-thesis-8026.
3. Hashemizadeh, S., Biancoli, A. & Damjanovic, D. Symmetry breaking in hexagonal and cubic polymorphs of $BaTiO_3$. *J. Appl. Phys* **119**, 094105 (2016).
4. Biancoli, A., Fancher, C. M., Jones, J. L. & Damjanovic, D. Breaking of macroscopic centric symmetry in paraelectric phases of ferroelectric materials and implications for flexoelectricity. *Nature Materials* **14**, 224–229 (2015).
5. Zhou, L., Vilarinho, P. M. & Baptista, J. L. Dependence of the Structural and Dielectric Properties of $Ba_{1-x}Sr_xTiO_3$ Ceramic Solid Solutions on Raw Material Processing. *J. Europ. Ceram. Soc.* **19**, 2015 (1999).
6. Lemanov, V. V., Smirnova, E. P., Syrnikov, P. P. & Tarakanov, E. A. Phase transitions and glasslike behavior in $Sr_{1-x}Ba_xTiO_3$. *Physical Review B* **54**, 3151–3157 (1996).
7. Hashemizadeh, S. & Damjanovic, D. Nonlinear dynamics of polar regions in paraelectric phase of $(Ba_{1-x},Sr_x)TiO_3$ ceramics. *Applied Physics Letters* **110**, 192905 (2017).
8. Darlington, C. N. W. & Cernik, R. J. The ferroelectric phase transition in pure and lightly doped barium titanate. *J. Phys.: Condensed Matter* **3**, 4555 (1991).
9. Hagemann, H.-J. Loss mechanisms and domain stabilisation in doped $BaTiO_3$. *J. Phys. C: Solid State Phys.* **11**, 3333–3344 (1978).
10. Lambert, M. & Comes, R. The chain structure and phase transition of $BaTiO_3$ and $KNbO_3$. *Solid State Com.* **7**, 305 (1969).

11. Schaffer, B. *DigiScan Stack Acquisition tool*. (2015).
12. Mitchell, D. *Stack Alignment*. (DigitalMicrograph, 2015).
13. Jones, L. *et al.* Smart Align—a new tool for robust non-rigid registration of scanning microscope data. *Advanced Structural and Chemical Imaging* **1**, 8 (2015).
14. De Backer, A., van den Bos, K. H. W., Van den Broek, W., Sijbers, J. & Van Aert, S. StatSTEM: An efficient approach for accurate and precise model-based quantification of atomic resolution electron microscopy images. *Ultramicroscopy* **171**, 104–116 (2016).
15. Zhou, D. *et al.* Sample tilt effects on atom column position determination in ABF–STEM imaging. *Ultramicroscopy* **160**, 110–117 (2016).
16. Kim, Y.-M., Pennycook, S. J. & Borisevich, A. Y. Quantitative comparison of bright field and annular bright field imaging modes for characterization of oxygen octahedral tilts. *Ultramicroscopy* **181**, 1–7 (2017).
17. Gao, P. *et al.* Picometer-scale atom position analysis in annular bright-field STEM imaging. *Ultramicroscopy* **184**, 177–187 (2018).
18. Koch, C. Determination of core structure periodicity and point defect density along dislocations. (Arizona State University, 2002).
19. Levin, I., Krayzman, V. & Woicik, J. C. Local structure in perovskite (Ba,Sr)TiO_3: Reverse Monte Carlo refinements from multiple measurement techniques. *Phys. Rev. B* **89**, 024106 (2014).
20. Serrano, S., Duque, C., Medina, P. & Stashans, A. Oxgen-vacancy defects in $PbTiO_3$ and $BaTiO_3$ crystals: a quantum chemical study. *Proc. SPIE, Advanced Organic and Inorganic Optical Materials* **5122**, 287–294 (2003).
21. Islam, Md. A., Momin, Md. A. & Nesa, M. Effect of Fe doping on the structural, optical and electronic properties of $BaTiO_3$: DFT based calculation. *Chinese Journal of Physics* **60**, 731–738 (2019).
22. Maldonado, F., Jácome, S. & Stashans, A. Codoping of Ni and Fe in tetragonal $BaTiO_3$. *Computational Condensed Matter* **13**, 49–54 (2017).
23. Stashans, A. & Castillo, D. Simulation of iron impurity in $BaTiO_3$ crystals. *Physica B* **404**, 1571–1575 (2009).
24. LeBeau, J. M. & Stemmer, S. Experimental quantification of annular dark-field images in scanning transmission electron microscopy. *Ultramicroscopy* **108**, 1653 (2008).
25. Rojac, T. *et al.* Domain-wall conduction in ferroelectric $BiFeO_3$ controlled by accumulation of charged defects. *Nature Materials* **16**, 322 (2017).
26. Aktas, O., Carpenter, M. A. & Salje, E. K. H. Polar precursor ordering in $BaTiO_3$ detected by resonant piezoelectric spectroscopy. *Applied Physics Letters* **103**, 142902 (2013).
27. Salje, E. K. H. *et al.* Elastic excitations in $BaTiO_3$ single crystals and ceramics: Mobile domain boundaries and polar nanoregions observed by resonant ultrasonic spectroscopy. *Phys. Rev. B* **87**, 014106 (2013).
